# Management of Plant Physiology with Beneficial Bacteria to Improve Leaf Bioactive Profiles and Plant Adaptation under Saline Stress in *Olea europea* L.

**DOI:** 10.3390/foods9010057

**Published:** 2020-01-07

**Authors:** Estrella Galicia-Campos, Beatriz Ramos-Solano, Mª. Belén Montero-Palmero, F. Javier Gutierrez-Mañero, Ana García-Villaraco

**Affiliations:** Universidad San Pablo-CEU Universities, Facultad de Farmacia, Ctra Boadilla del Monte km 5.3, 28668 Boadilla del Monte, Madrid, Spain; e.galicia@usp.ceu.es (E.G.-C.); bramsol@ceu.es (B.R.-S.); mariabelen.monteropalmero@ceu.es (M.B.M.-P.); jgutierrez.fcex@ceu.es (F.J.G.-M.)

**Keywords:** olive, salinity, osmolytes, adaptation, secondary metabolism, plant growth promoting rhizobacteria (PGPR), net photosynthesis, oleuropein, water use efficiency (WUE)

## Abstract

Global climate change has increased warming with a concomitant decrease in water availability and increased soil salinity, factors that compromise agronomic production. On the other hand, new agronomic developments using irrigation systems demand increasing amounts of water to achieve an increase in yields. Therefore, new challenges appear to improve plant fitness and yield, while limiting water supply for specific crops, particularly, olive trees. Plants have developed several innate mechanisms to overcome water shortage and the use of beneficial microorganisms to ameliorate symptoms appears as a challenging alternative. Our aim is to improve plant fitness with beneficial bacterial strains capable of triggering plant metabolism that targets several mechanisms simultaneously. Our secondary aim is to improve the content of molecules with bioactive effects to valorize pruning residues. To analyze bacterial effects on olive plantlets that are grown in saline soil, photosynthesis, photosynthetic pigments, osmolytes (proline and soluble sugars), and reactive oxygen species (ROS)-scavenging enzymes (superoxide dismutase-SOD and ascorbate peroxidase-APX) and molecules (phenols, flavonols, and oleuropein) were determined. We found photosynthetic pigments, antioxidant molecules, net photosynthesis, and water use efficiency to be the most affected parameters. Most strains decreased pigments and increased osmolytes and phenols, and only one strain increased the antihypertensive molecule oleuropein. All strains increased net photosynthesis, but only three increased water use efficiency. In conclusion, among the ten strains, three improved water use efficiency and one increased values of pruning residues.

## 1. Introduction

The traditional olive production system in the Mediterranean was developed in dry, farmed areas with trees spaced from 25 to 60 feet (7.6–18.3 m) apart, giving 12 to 70 trees/acre (30–173 trees/ha) [1]. Thus, olive trees are often under severe water deficit combined with high temperatures and high light intensities during the summer season. Moreover, the traditional olive production system in dry farms has many disadvantages such as low yields, delays before full production (15–40 years) and a very inefficient non-mechanical harvest [1]. In recent decades, the cultivation of the Spanish olive has undergone major technological changes associated with high-density or super high-density production systems, such as a reduction in the number of olive varieties, an increase in the density of the new plantations, an improvement of the harvesting machinery or orchard irrigation [2]. Therefore, this change from dry, farmed areas to irrigated cultivation has placed water stress or high salt concentration, as one of the main problems that olive cultivation is currently facing. In this agronomic framework, in which olive trees require irrigation, the possibility of producing in areas with high salt concentration or reduced water supply represents an important economic advantage.

Although olive resists a high degree of drought stress, the acclimation ability of olive plants to adjust to water deficit includes two mechanisms: avoidance and tolerance [3,4]. Among the innate acclimation mechanisms of plants are morphological and physiological leaf alterations; reduction of leaf size and number; biosynthesis and accumulation of compatible solutes (amino acids, proteins, sugars, methylated quaternary ammonium compounds, and organic acids); hormonal balance alteration (abscisic acid-ABA and ethylene); increase in ion efflux with high-affinity antiporters (salt overly sensitive-SOS1 and high-affinity potassium transporters-HKT); maintenance of reactive oxygen species (ROS) homeostasis, and decrease of photosynthetic rates [5,6]. Photosynthetic rates decrease mostly due to stomatal closure, but as water stress becomes severe, the inactivation of photosynthetic activity could be due not only to stomatal restrictions, but also to non-stomatal factors related to inhibition of primary photochemistry and electron transport in chloroplasts [7] as well as to the increase in reactive oxygen species (ROS) levels. When the absorbed light energy is not fully used by photosynthesis, it deviates to molecular oxygen, which is abundant in the chloroplasts [8] leading to ROS formation. ROS are highly reactive oxygen species constantly generated by cell organelles as a metabolic by-product; they function as signaling molecules, but their production is spiked upon stress, and plant normal metabolism is seriously disrupted [9].

Plants have a complex antioxidant system to cope with ROS involving enzymes and molecules [10]. The major enzymatic scavengers of ROS are superoxide dismutase (SOD), ascorbate peroxidase (APX), and catalase (CAT) [11]. In addition, plants contain several low-molecular-weight antioxidants, such as ascorbate, glutathione, and phenolic compounds, which are water-soluble, and tocopherol and carotenoids, which are lipid-soluble [12]. Although studies on the enzymatic antioxidant system of olive trees under water deficit have demonstrated that antioxidant enzymes play a major role in protecting olive leaf tissues against oxidative stress [13,14,15], the role of phenolic compounds on the water stress tolerance of olive trees has received limited attention [5].

As sessile organisms, olive plants have an active secondary metabolism to improve their adaptation to biotic and abiotic stress conditions [16]. The most characteristic secondary metabolites present in olive trees are iridoids, triterpenes, and phenolic compounds. These metabolites accumulate preferentially in leaves and their beneficial effects as antihypertensive for human health due to the coordinated effects of iridoids (oleuropein, oleacein, and ligustroside) and triterpenes (oleanolic acid) have been previously demonstrated [17,18,19]; their antitumor potential has also been reported [20].

As water stress is a relevant problem, plants have many innate mechanisms that regulate adaptation to stress. Biotechnological attempts to improve adaptations to water deficit with genetic modifications target the overexpression of different genes, as in *Arabidopsis* [21] or cereals, such as rice [22]. In woody plants like the olive tree with such a large cropping surface, genetic modification is not the best choice to improve adaptation. An alternative to genetic modification addressing several targets is the plants’ natural associates, soil microorganisms, especially, beneficial strains termed plant growth-promoting rhizobacteria (PGPR).

The term plant growth-promoting rhizobacteria was coined by Kloepper et al. in 1980 [23] to refer to free-living bacteria that inhabit the rhizosphere, which is the soil closely related to the roots. The mechanisms by which these PGPR improve plant fitness have been largely reviewed [24,25]; PGPR affects plant external factors such as nutrient mobilization or biocontrol of soil microorganisms, or alter internal metabolism by affecting endogenous hormonal balance or systemic induction of metabolism at different levels, like photosynthesis or secondary metabolism. Thus, the role of beneficial rhizobacteria to trigger secondary metabolism appears as a promising alternative to increase the levels of bioactive secondary metabolites [26,27,28,29], protect against biotic stress [30] and other frequent situations in agriculture [31]. More precisely, protection against salt stress can be enhanced by beneficial rhizobacteria by boosting the ROS-scavenging system, increasing compatible solute concentration, such as proline or soluble sugars, or improving photosynthesis and water use efficiency [6].

Therefore, the use of beneficial rhizobacteria capable of modulating secondary metabolism pathways of plants appears as a biotechnological tool with great potential for this purpose. The application of beneficial strains to improve adaptation to abiotic stress has been widely shown for different species, either woody or herbaceous crops, targeting many mechanisms simultaneously [6] and therefore, with great chances of success. To our knowledge, no studies have been undertaken specifically on olive plants to improve adaptation to salt stress with beneficial rhizobacteria, paying specific attention to bioactive molecules accumulated in leaves; furthermore, if bioactives accumulate in leaves, pruning residues can be transformed into a valuable side product, to obtain enriched extracts with antihypertensive potential. Our rationale is that inoculating the olive trees with beneficial rhizobacteria would simultaneously trigger secondary metabolism pathways as well as other mechanisms also involved in abiotic stress adaptation. We selected 10 bacterial strains from a previous screening in *Pinus* rhizosphere [32] to evaluate their ability to improve olive tree adaptation to salt stress and enhance bioactive contents. To achieve this objective, photosynthesis was measured after 12 months of inoculations on plantlets grown in high saline conditions, photosynthetic pigments, osmolites (soluble sugars, proline), ROS scavenging enzymes (SOD, APX), and antioxidant molecules (phenols, flavonols, and oleouropein) were analyzed as markers of the overall fitness of the plant.

## 2. Materials and Methods

### 2.1. Beneficial Strains and Olive Tree Variety

The 10 beneficial strains (L79, L81, L56, L24, L62, L36, G7, L44, K8, and H47) assayed in this study were isolated from the rhizosphere of *Pinus pinea* and *P. pinaster* [32]. They were able to produce siderophores (L79, L81, G7, H47), auxins (L56, L24, L44), auxins and siderophores (L62, L36) or auxins and degrade ACC (K8). Except for L62, a Gram-positive non-esporulated rod, all other strains are Gram-positive esporulated bacilli.

*Olea europea* (L) var. Arbosana plantlets were used for the study. Plantlets were bought from a commercial producer.

### 2.2. Inocula Preparation and Delivery to Plants

Bacterial strains were maintained at −80 °C in nutrient broth with 20% glycerol. Inocula were prepared by streaking strains from −80 °C onto plate count agar (PCA) plates, incubating plates at 28 °C for 24 h. Then, they were grown in Luria Broth liquid media (LB) or nutrient broth (only L62) under shaking (1000 rpm.) at 28 °C for 24 h to obtain a 2 × 10^9^ cfu/mL inoculum.

These cultures were adjusted to 1 × 10^8^ cfu/mL and 500 mL were root-inoculated every 15 days from October 2017 to October 2018.

### 2.3. Experimental Design

Six-month olive plantlets were transplanted into 5 L pots with soil from the Guadalquivir Marshes. Plants were arranged in lines on an experimental plot within the marshes (37°06′34.5′′ N, 6°20′22.7′′ W); pot position was changed every two weeks to avoid side-effects. Plants were watered every 15 days. The electric conductivity of water was 8.20 dS/m and of soil it was 6.07 dS/m.

Bacteria were root-inoculated by soil drench every 15 days from October 2017 to October 2018; so plants received 500 mL of water every week, alternating inoculum and water. Six plants per treatment were inoculated, being one bacterial strain a treatment. Samples were taken in October 2018 and photosynthesis was measured (fluorescence and CO_2_ fixation). Leaves were powdered in liquid nitrogen and stored at −60 °C till analysis. Photosynthetic pigments were determined as well as their antioxidant capacity, analyzing both the enzymatic and non-enzymatic apparatus. Superoxide dismutase (SOD) and ascorbate peroxidase (APX) activities were determined as indicators of the enzyme apparatus, and total phenols, flavonols and oleuropein, as indicators of the non-enzymatic pool. The osmoprotective effect was evaluated by analyzing compatible solutes (proline and soluble sugars).

### 2.4. Photosynthesis (Chlorophyll Fluorescence)

Photosynthetic efficiency was determined through the chlorophyll fluorescence emitted by photosystem II. Chlorophyll fluorescence was measured with a pulse amplitude modulated (PAM) fluorometer (Hansatech FM2, Hansatech, Inc., UK). After dark-adaptation of leaves, the minimal fluorescence (Fo; dark-adapted minimum fluorescence) was measured with a weak modulated irradiation (1 μmol m^−2^ s^−1^). Maximum fluorescence (Fm) was determined for the dark-adapted state by applying a 700 ms saturating flash (9000 μmol m^−2^ s^−1^). The variable fluorescence (Fv) was calculated as the difference between the maximum fluorescence (Fm) and the minimum fluorescence (Fo). The maximum photosynthetic efficiency of photosystem II (maximal PSII quantum yield) was calculated as Fv/Fm. Immediately, the leaf was continuously irradiated with red-blue actinic beams (80 μmol m^−2^ s^−1^) and equilibrated for 15 s to record Fs (steady-state fluorescence signal). Following this, another saturation flash (9000 μmol m^−2^ s^−1^) was applied and then Fm’ (maximum fluorescence under light-adapted conditions) was determined. Other fluorescent parameters were calculated as follows: the effective PSII quantum yield ΦPSII = (Fm’ − Fs)/Fm’ [33]; and the non-photochemical quenching coefficient NPQ = (Fm − Fm’)/Fm’. All measurements were carried out in the 6 plants of each treatment.

### 2.5. Photosynthesis (CO_2_ Fixation)

Net photosynthetic rate, (Pn) (mmol CO_2_/m^2^), transpiration rate, E (mmol/m^2^ s) and stomatal conductance, C (mmol/m^2^ s) were measured with a portable photosynthetic open-system (CI-340, CID, Camas, WA, USA) [34].

Water use efficiency (WUE) was calculated as net photosynthesis (Pn) divided by transpiration (E) as an indicator of stomatal efficiency to maximize photosynthesis minimizing water loss due to transpiration.

### 2.6. Photosynthetic Pigments: Chlorophylls and Carotenoids

Extraction was done according to [35]. One hundred mg of leaves powdered in liquid nitrogen was dissolved in 1 mL of acetone 80% (*v*/*v*), incubated overnight at 4 °C and then centrifuged 5 min at 10,000 rpm in a Hermle Z233 M-2 centrifuge. One mL of acetone 80% was added to the supernantant and was mixed with a vortex. Immediately afterward, absorbance at 647, 663, and 470 nm was measured on a Biomate 5 spectrophotometer to calculate chlorophyll a, chlorophyll b, and carotenoids (xanthophylls + carotenes) using the formulas indicated below [35,36].

Chl a (µg/g FW) = [(12.25 × Abs_663_) − (2.55 × Abs_647_)] × V(mL)/weight (g).

Chl b (µg/g FW) = [(20.31 × Abs_647_) − (4.91 × Abs_663_)] × V(mL)/weight (g).

Carotenoids (µg/g FW) = [((1000 × Abs_470_) − (1.82 × Chl a) − (85.02 × Chl b))/198] × V(mL)/weight (g).

Tubes were protected from light throughout the whole process.

### 2.7. Enzymatic Antioxidants: Superoxide Dismutase (SOD) and Ascorbate Peroxidase (APX)

Before assessing enzymatic activities, soluble proteins were extracted by suspending 100 mg of powder in 1 mL of potassium phosphate buffer 0.1 M, pH 7.0, containing 2 mM phenylmethylsulfonyl fluoride (PMSF). After sonication for 10 min and centrifugation for 10 min at 14,000 rpm, the supernatant was aliquoted, frozen in liquid nitrogen and stored at −80 °C for further analysis of APX, SOD, and proteins. All the above operations were carried out at 0–4 °C.

To measure the amount of total protein from plant extract, 250 µL of Bradford reagent and 5 µL of sample and BSA dilutions were inoculated in ELISA 96 well plates and incubated for 30 min at room temperature and measured using a plate reader at an absorbance of 595 nm. A calibration curve was constructed from commercial BSA dilutions expressed in milligrams. The protein units were expressed as mg/µL.

APX was measured by the method of Garcia-Limones [37]. The reaction mixture consisted of 50 mM potassium phosphate buffer, pH 7.0, 0.25 mM sodium ascorbate, 5 mM H_2_O_2_ and 100 μL of enzyme extract in a final volume of 1.2 mL. Adding H_2_O_2_ started the reaction and the oxidation of ascorbate was determined by the decrease in A290. The extinction coefficient of 2.8 mM^−1^ cm^−1^ was used to calculate activity. One unit of APX activity is defined as the amount of enzyme that oxidizes 1 mmol min^−1^ of ascorbate under the above assay conditions.

SOD activity was determined following the specifications of the SOD activity detection kit (SOD Assay Kit-WST, Sigma-Aldrich, Darmstadt, Germany). With this method, xanthine is converted to superoxide radical ions, uric acid, and hydrogen peroxide by xanthine oxidase (XO). Superoxide reacts with WST1 to generate a product that absorbs at around 440 nm. SOD prevents the reduction of WST1 to WST-1formazan, thus reducing the absorption at 440 nm, which is proportional to SOD activity; the rate of the reduction of WST1 with O_2_ is linearly related to the xanthine oxidase (XO) activity. The unit used for this activity was: % inhibition of WST reduction.

### 2.8. Osmolites: Proline and Soluble Sugars

An ethanolic extraction was prepared from 0.25 g of powder and 5 mL of ethanol 70% (*v*/*v*) incubated at 100 °C for 20 min.

For proline determination 1 mL of ninhydrin reagent freshly prepared (1 g of ninhydrin dissolved in 60 mL of acetic acid, 20 mL of ethanol and 20 mL of water) was mixed with 0.5 mL of the plant ethanol extract and heated at 95 °C for 20 min. Finally, absorbance at 520 nm was measured. Results are expressed as µmol/g.

A soluble sugars determination was performed following Yemm and Willis [38]. Briefly, the following reaction was prepared: 3 mL of the reactive (200 mg of antrone + 100 mL of 72% sulfuric acid) and 0.1 mL of the plant ethanol extract. The reaction was incubated in a bath at 100 °C for 10 min. Once it was cold, absorbance was measured at 620 nm. To calculate soluble sugar concentration the following equation was used:

µg/g = [(Abs_620_ − 0.016)/0.02]/weight (g)/1000

### 2.9. Total Phenols and Flavonols

Leaf extracts were prepared from 0.25 g of leaves (powdered in liquid nitrogen) in 2.25 mL methanol 80%, sonicated for 10 min and centrifuged for 5 min at 5000 rpm.

Total phenols were quantitatively determined with Folin-Ciocalteu agent (Sigma. Aldrich, St Louis, MO, USA) by a colorimetric method described by Xu and Chang [39], with some modifications, gallic acid was used as standard (Sigma-Aldrich, St Louis, MO, USA). Twenty microlitres of extract were mixed with 0.250 mL of Folin-Ciocalteu 2 N and 0.75 mL of Na_2_CO_3_ 20% solution. After 30 min at room temperature, absorbance was measured at 760 nm. A gallic acid calibration curve was made (r = 0.99). Results are expressed in mg of gallic acid equivalents per 100 g of fresh weight (FW).

Total flavonols were quantitatively determined through the test described by Jia et al. [40], using catechin as standard (Sigma-Aldrich, St Louis, MO, USA). One milliliter of the extract was added to a flask of 10 mL with 4 mL of distilled water. After that 0.3 mL of NaNO_2_ 5%, and 0.3 mL of AlCl_3_ 10% were added after 5 min. One minute later, 2 mL of NaOH 1 M were added, and distilled water was added util 10 mL of total volume. The solution was mixed and measured at 510 nm. A catechin calibration curve was made (r = 0.99). Results are expressed as mg of catechin equivalents per 100 g of fresh weigh (FW).

### 2.10. Oleuropein Extraction and TLC Analysis

Oleuropein was determined according to the European Pharmacopoeia. One gram of the powdered samples was extracted with 10 mL of methanol under reflux for 15 min. After cooling, samples were filtered and 10 µL was loaded as a band on a TLC silica gel plate; the reference solution contained 10 mg of oleuropein and 1 mg of rutoside trihydrate in 1 mL of methanol. Plates were incubated on a chromatography tank and allowed to develop over a path of 10 cm, being the mobile phase water/methanol/methylene chloride (1.5:15:85 *v*/*v*/*v*). Plates were dried in air. Detection of oleuropein was done by spraying with vanillin sulphuric acid reagent after followed by heating for 5 min at 100–105 °C; the brownish-green zone appeared in the middle of the plate was oleuropein and a brownish-yellow zone near the application point was rutoside.

Quantification was done with the image analysis program Quantity One v4.6.8 (Biorad, CA, USA), based on the density and concentration of the oleuropein spot from the reference sample.

### 2.11. Statistics

A Principal Components Analysis (PCA) with all the parameters measured for the ten strains was performed with Canoco^TM^ for Windows v.4.5 software (Microcomputer power, Ithaca, USA) [41]. Scaling was performed withinter-species correlation and was achieved dividing by the standard deviation.

To evaluate treatment effects, one way ANOVA (Statpgraphcis Centurion XVIII) were performed for each of the variables. When significant differences appeared (*p* < 0.05), the LSD test (least significant difference) from Fisher was used.

## 3. Results

**Figure 1:**
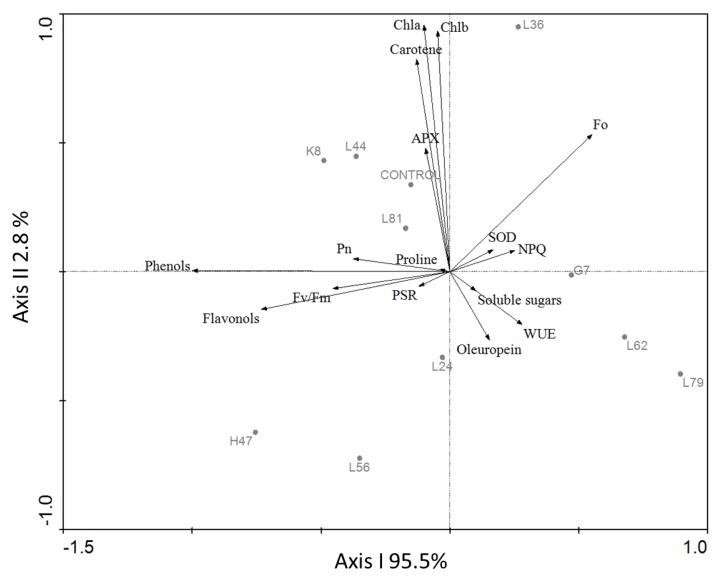
Ordination provided by the principal components analysis (PCA) performed with all the parameters measured for the ten strains. APX, ascorbate peroxidase; SOD, superoxide dismutase; NPQ, non-photochemical quenching coefficient; WUE, Water Use Efficiency. Percentages in the axis correspond to the variance absorbed by each of these two first axes.

Three main groups can be defined in the ordination provided by principal component analysis (PCA) (Figure 1). The group in the upper part of axis I (dotted line) includes bacteria L36, K8, L44, and L81 and is mainly influenced by photosynthetic pigments concentration and, secondarily, by APX and SOD activities as shown by the length of the vectors. A second group including K8, L44, L81, H47, and L56 (black dashed line) on the left of axis I, can be defined based on phenols and flavonols concentration. A third group formed by L56, L24, and L62 (grey dashed line), is determined by oleuropein concentration and water use efficiency (WUE).

**Figure 2:**
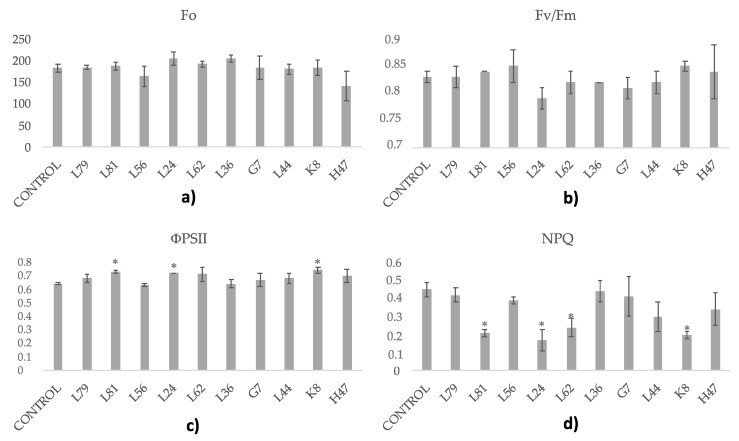
Photosynthetic parameters related to photosystems and light reactions. (**a**) Minimal fluorescence after 20-min dark-adaptation (Fo). (**b**) Maximal PSII quantum yield (Fv/Fm), (**c**) effective PSII quantum yield (ΦPSII) and (**d**) non-photochemical quenching coefficient (NPQ) measured in olive tree plants treated with the ten strains and the non-inoculated control. For each treatment and parameter average value ± standard error value (*n* = 6) is presented. Asterisks (*) represent significant differences with the control according to the LSD test (*p* < 0.05).

**Figure 3:**
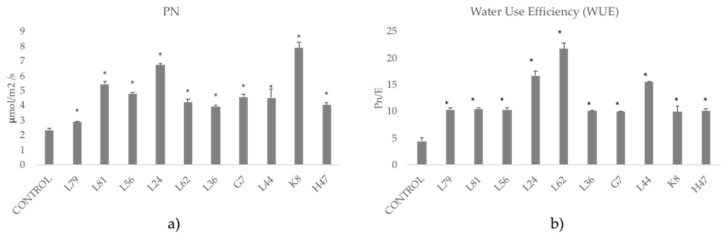
Photosynthetic parameters related to C fixation measured in olive tree plants treated with the ten strains. (**a**) Net photosynthesis (PN) measured as the CO_2_ fixed by the leaves (μmol CO_2_ /m^2^ s). (**b**) Water Use Efficiency (WUE) calculated as PN divided by transpiration rate (μmolH_2_O /m^2^ s). Average values of the replicates with standard error bars are represented (*n* = 6). Asterisks (*) represent significant differences with the control according to the LSD test (*p* < 0.05).

All assayed strains modified photosynthetic parameters (Figure 2), however, Fv/Fm was within normal values (0.82–0.85) and was not significantly affected by any strain. L81, L24, and K8 significantly increased photochemical quenching and all strains except L79 and L36 decreased energy dissipation (NPQ). All of them significantly increased net photosynthesis in terms of CO_2_ fixation, with an outstanding performance of K8 and L24 that caused 3-fold increases, while all others were in the range of 2-fold increases. Water use efficiency, calculated as the value of net photosynthesis divided by the transpiration rate, was also significantly increased by all strains, with an outstanding performance of L62 that caused a 5-fold increase on WUE, and L24 and L44 in second place, in the range of a 3.5 increase on WUE (Figure 3).

**Figure 4:**
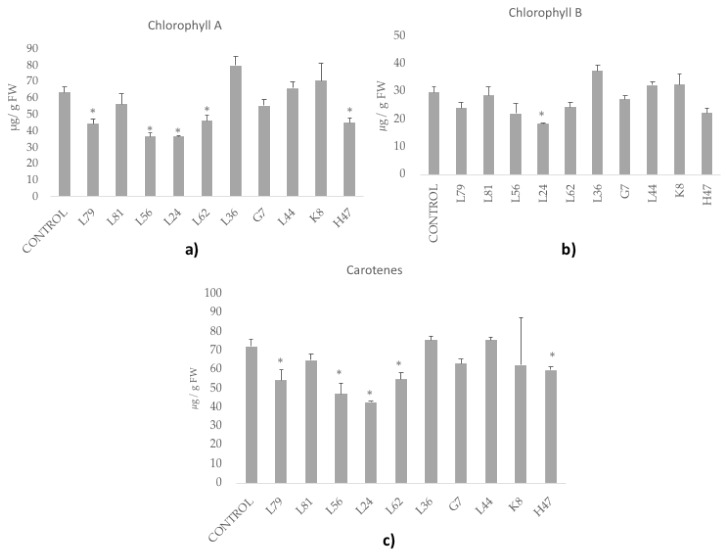
Photosynthetic pigments concentration (µg/g FW). (**a**) Chlorophyll a, (**b**) Chlorophyll b and (**c**) Carotenoids were measured in olive tree leaves treated with the ten strains. For each treatment and parameter average value ± standard error value is presented (*n* = 6). Asterisks (*) represent significant differences with the control according to the LSD test (*p* < 0.05).

As regards to photosynthetic pigments (Figure 4), controls had around 63 mg/g chlorophyll a, 29.69 chlorophyll b, and 72.3 mg/g carotenes. Most strains maintained or decreased the amount of chlorophylls, except for strain L36 that increased chlorophylls and K8 that increased chlorophyll a; none modified the chlorophyll a/b ratio. The general trend was to lower carotene concentration, except for L36 and L44.

**Figure 5:**
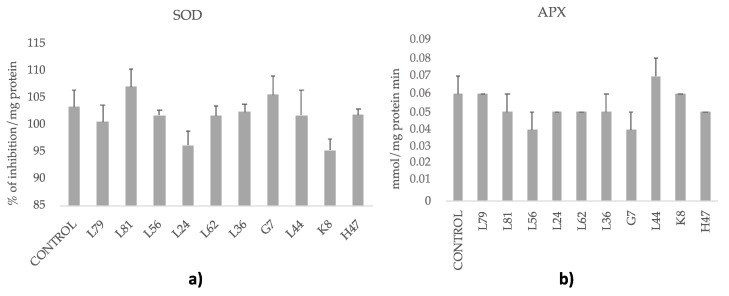
Enzyme activities related to oxidative stress. (**a**) Superoxide dismutase (SOD), (**b**) Ascorbate peroxidase (APX) activities measured in olive tree leaves treated with the ten strains. Enzymatic activities were calculated as mmol/mg protein min (for APX) and % of inhibition/mg protein (for SOD). For each treatment and parameter average value ± standard error value is presented (*n* = 6). There are no significant differences according to the LSD test (*p* < 0.05).

As for the enzymatic antioxidant systems (Figure 5), only L81 increased the activity of SOD and L44 that of APX, while no changes or slight decreases were induced by the other 8 strains.

**Figure 6:**
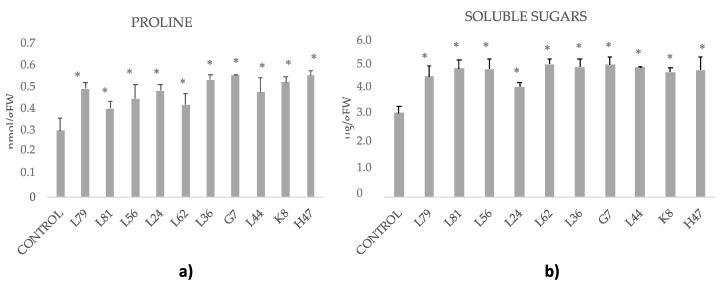
(**a**) Proline (nmol/g fresh weight) and (**b**) soluble sugars concentration (μmol/g fresh weight) measured in olive tree leaves treated with the ten strains. Average values of the replicates with standard error bars are represented (*n* = 6). Asterisks (*) represent significant differences with the control according to the LSD test (*p* < 0.05).

Soluble sugars in controls were 3.77 mg/g and proline contents 0.37 µmol/g (Figure 6). Soluble sugars contents were significantly increased by all treatments, ranging from 10% (L24) to 30% (L62 (Figure 6a)). Similarly to soluble sugars, proline was significantly increased by most strains ranging from 20% (L56) to 50% (G7, H47); only L81 and L62 showed similar proline contents than controls (Figure 6b).

**Figure 7:**
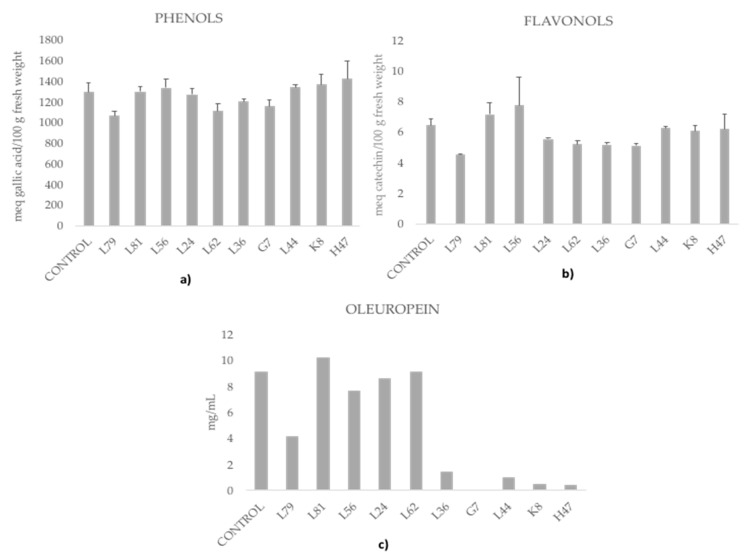
Bioactives. (**a**) Phenols (meq gallic acid/100 g fresh weight), (**b**) flavonols (meq catechin/100 g fresh weight) and (**c**) oleuropein (mg/mL) concentration measured in olive tree leaves treated with the ten strains. For each treatment and parameter average value ± standard error value is presented. There are no significant differences according to the LSD test (*p* < 0.05).

As for bioactives (Figure 7), controls had high phenols concentration (1295 meq gallic acid/100 g FW) and low flavonols (6.46 meq catechin/100 g FW), and 9.11 mg oleuropein/g. Strains L81 and L56 increased the concentration of phenols and flavonols in leaves and L44, K8, and H47 only that of phenols. Only L81 increased oleuropein concentration (12%), L62 did not affect it and most strains decreased the amount of oleuropein, a group with minor decreases (L56, L24, L79) and another group with major decrease.

## 4. Discussion

In this study, the ability of the ten beneficial rhizobacteria assayed to modify plant physiology of olive plantlets growing in pots with high electric conductivity of soil and water when root-inoculated has been evidenced. According to Chartzouaskis [42], values of water (8.20 dS/m) and soil (6.07 dS/m) electric conductivity, indicate severe salinity for olive trees in the present study. In these harsh conditions, all strains are able to trigger plant adaptative metabolism, improving net photosynthesis and mainly affecting osmolite concentration. The positive effect of these strains was expected since the original screening rendered several beneficial strains, two of which (L62 and L81) are also tested in this experiment [30,32].

In general, the effects of bacteria on plant metabolism and physiology target photosynthetic pigments, photosynthesis, and osmolites [8] as revealed by the principal components analysis (PCA), a multivariate analysis that provides an ordination of the samples based on their similarity considering all the variables analyzed. In this ordination (Figure 1), samples are grouped mainly due to the differential effects of bacteria in those three variables. The group in the upper part of axis I (dotted line) includes bacteria that increase (L36 and K8) or maintain (L44 and L81) photosynthetic pigments. The position of these two bacteria (L44 and L81) in the ordination is also determined by the effect they have in APX and SOD, respectively. All the other bacteria decreased pigment concentration. Bacteria in the second group (black dashed line), K8, L44, L81, H47, and L56, increase leaf phenol concentrations, and L81 and L56 also increase flavonols. A third group formed by L56, L24, and L62 (grey dashed line) includes the three bacteria that maintain oleuropein concentration similar to control plants, while the other bacteria reduce this concentration, except for L81, the only strain that increases oleuropein concentration in leaves. Finally, L24 and L62 induced the highest water use efficiency in the plants. Therefore, all these parameters are differentially affected by the different strains while proline, soluble sugars concentration, and net photosynthetic rate are similarly triggered by all bacteria [6], as can be noticed by the shorter length of vectors.

Under mild and moderate water stress, photosynthetic rate decreases in olive plants mostly due to stomatal closure [43]. However, as water stress becomes severe, the inactivation of photosynthetic activity could be due not only to stomatal closure but also to non-stomatal factors related to inhibition of primary photochemistry and electron transport in chloroplasts [7]. A decrease in chlorophylls under salt stress has been explained by pigment destruction after the ROS peak [9]. However, if this was the case, photosynthesis would be impaired due to cell membrane alterations and lack of pigments. Interestingly, our data show increased effective PSII quantum yield (ΦPSII) (L81, L24 and K8), and lower NPQ, suggesting that bacteria are triggering an innate plant protective mechanism against the excess of light entering the system, and making better use of the energy fixed.

Under salt stress conditions olive leaves become thicker [44] compromising CO_2_ diffusion to chloroplasts [45,46]. Photosynthesis is reduced under saline stress in olive trees [44,47,48], but with different effects on the CO_2_ assimilation rate depending on salt concentration. Interestingly, all strains increased net photosynthesis, consistent with the reported modification of carbohydrate production and sink utilization that leads to downregulate feedback photoinhibition and boost plant energy metabolism [49], probably providing C scaffoldings for secondary metabolites and osmolyte synthesis and accumulation. Despite the significant increase in C fixation induced by all strains, water use efficiency (WUE) was different, with a striking two-fold increase for most strains except for L62, which showed a 4-fold increase, and L44 and L24, showing a 3-fold increase (Figure 3). This indicates a strong improvement in plant fitness in a high saline environment, suggesting activation of protective systems and highlighting the different mechanisms involved in adaptation to harsh conditions and supporting the multivariate solution provided by PGPR [6].

There is a demonstrated relationship between compatible solutes and photosynthesis. All strains increased concentration of compatible solutes as a protective mechanism, since they sequester water molecules, protect cell membranes and protein complexes, and allow the metabolic machinery to continue functioning [8]. Consistent with our data, carbohydrates are the most common solutes accumulated in olive tree tissue under water deficit conditions [6,50], and all strains significantly increased them, being thus a primary defense mechanism [49]. The close relationship between net photosynthetic rate and proline content reported by BenAhmed et al. [13], is consistent with our data, as proline was increased by all except for L81 and L62, confirming the important role of this osmolyte in the maintenance of photosynthetic activity and plant homeostasis. The different behaviors suggest the involvement of other factors, as L81 and L62 performed outstandingly.

Bacterial strains modify differently innate plant mechanisms to cope with ROS [29,51]; while the enzyme ROS scavenging system is hardly modified by bacteria, phenolic compounds are. The enzyme system is probably in its maximum natural activation, which cannot be further increased by bacteria; however, L81 is still able to significantly enhance SOD activity. Although studies on the enzymatic antioxidant system of the olive tree under water deficit have demonstrated that antioxidant enzymes play a major role in protecting olive leaf tissue against oxidative stress [13,14,15], limited attention has been given to the effect of phenolic compounds on water stress tolerance. Phenolic compounds are constitutively present in all higher plants. However, phenylpropanoid metabolism is often induced when plants are exposed to a wide range of environmental stresses [52], including bacteria [26,53]. In view of our results, abiotic stress as well as beneficial rhizobacteria modified the antioxidant pools of the plants but in an uncoupled way. Some of the assayed strains increased total phenols and flavonols (L81 and K8), and oleuropein (only L81), while another group decreased concentrations of these metabolites (L79, L24, L62, and G7). Among the first group, L81 increased SOD enzyme activity. From the second group, L79 increased SOD and L24 decreased both SOD and APX enzymatic activities, which suggests either higher oxidative stress as a consequence or other mechanisms to cope with ROS (Figure 5). The different influence of bacterial strains on ROS scavenging enzyme activity has been described before to be strain-specific [28]. García-Cristobal et al [28] reported the ability of a *Chryseobacterium* strain to enhance ROS scavenging enzymes activity in salt and pathogen stressed rice plants, while a *Pseudomonas* strain enhanced protection by increasing defensive enzymes, not ROS scavenging enzymes.

Under salt stress conditions, phenolic compounds produced in leaves increase [5,6,15]. However, in this work only five strains increased total phenolics concentration and only two significantly increased total flavonols, while others decreased them, reinforcing the species specificity between plant and bacteria [28,53], a receptor-mediated effect, hence highly specific. Irrespective of the final phenolics balance, all bacteria have altered this pathway, confirming the role of this pathway in adaptation; not only phenolics behave as antioxidants, but other derived molecules may also have this role [27,54]. Considering all this data together, it seems that bacteria are lowering photosynthetic pigments concentration, suggesting this effect as a mechanism to decrease oxidative stress due to photosynthesis, especially since the enzymatic antioxidant pool is not affected or even decreased, and bioactives are only increased by half of the strains. Finally, oleuropein, a bioactive molecule accumulated in leaves [18], with a proposed role as a protective molecule against biotic stress due to its potential as a cross-linking agent [54], is only increased under the influence of L81. Again, two strategies are depicted, either slightly lowering its concentration, or minimizing it reinforcing the hypothesis of each strain activating different mechanisms of plant adaptation.

Not only olive oil is obtained from olive trees; also solid residues obtained in considerable amounts during olive oil production and elaboration of table olives are of great concern in the Mediterranean area, as these by-products accumulate in large amounts. Great progress has been made to recycle these residues obtaining an economic profit, like obtaining activated carbon [55,56,57,58] or fuel for the generation of heat and electricity [59,60,61]. However, olive leaves, which are produced in large amounts, render scarce profits at present. Nevertheless, the market for natural ingredients and additives is rapidly growing, with such products obtaining high prices. Increased concentrations of phenolic compounds and especially oleuropein, with strong antihypertensive potential, reinforces the potential of olive leaves in the field of a circular economy. Furthermore, delivering beneficial strains to edible plants has improved beneficial effects for health as not only the targeted metabolites are increased, but there is a general physiological change that results in improved effects on health [53].

In summary, delivering beneficial strains improves adaptation to high saline conditions, mainly affecting osmolytes and improving net photosynthesis and water use efficiency. Interestingly, L81 differentially increases oleuropein constituting a good treatment to improve the potential of olive leaves for its antihypertensive effects. L62 is the one to improve WUE, which is especially good to improve plant adaptation to harsh conditions of low water availability.

## 5. Conclusions

In view of these data, it is evidenced that all bacterial strains improve plant adaptation increasing osmoprotection and net photosynthesis but they differentially affect the enzymatic and non-enzymatic antioxidant systems. Bacteria able to increase bioactive concentration and therefore potential benefits of olive leaves on health may also contribute to a circular economy, recycling pruning residues.
